# Electromyographic Activation of the Pectoralis Major and Triceps Brachii Muscles During Standard, Diamond, and Wide Hand Position Push-Ups

**DOI:** 10.3390/muscles5010018

**Published:** 2026-02-27

**Authors:** Konstantina Intziegianni, Epifanios Katsamis, Marcos Michaelides, Koulla Parpa

**Affiliations:** School of Science, University of Central Lancashire Cyprus (UCLan Cyprus), Pyla 7080, Cyprus; epifanios10@hotmail.com (E.K.); mmichaelides@uclan.ac.uk (M.M.); kparpa@uclan.ac.uk (K.P.)

**Keywords:** root mean square, consistency, concentric, eccentric, closed kinetic chain, normalization, neuromuscular activation

## Abstract

Studies examining the electromyographic activation of pectoralis major (PM) and triceps brachii (TB) muscles during push-ups of varied hand positions are limited, and findings are inconsistent. The aim was to investigate the electromyographic activation of PM and TB during standard, diamond, and wide hand position push-ups. Twenty young males performed six repetitions of each push-up variation while the electrical activity of PM and TB was recorded, averaged, and normalized to the peak root mean square (RMS) across repetitions for each push-up. RMS (mV) and normalized RMS (%) were calculated for each muscle, push-up variation, and contraction phase (eccentric/concentric). Two separate three-way ANOVAs with Bonferroni post hoc correction were used (*α* = 0.05). TB demonstrated statistically significantly higher RMS (mV) and normalized RMS (%) than PM (*p* < 0.05), in diamond, followed by standard and wide push-ups. A statistically significant higher activation of RMS (mV) was observed in concentric compared to eccentric (*p* < 0.05); however, after normalizing RMS (%), contraction phase had no effect (*p* > 0.05) and there was no significant three-way interaction (*p* > 0.05). Diamond push-ups elicited the highest relative activation for both the PM and TB. Normalized RMS revealed the consistency of muscle effort in both contraction phases, sustaining near-maximal activation regardless of hand position. These findings support adaptable training strategies, with potential applications in rehabilitation and strength training contexts.

## 1. Introduction

One of the most popular upper body strength exercises are push-ups which are used in rehabilitation [1] and strength training [2] contexts with the purpose of enhancing upper extremity muscular strength [3], neuromuscular control [4,5], and endurance [6]. Push-ups are closed kinetic chain exercises which involve movements of shoulder horizontal adduction/abduction and elbow flexion/extension [7]. The primary acting muscles are the pectoralis major and triceps brachii, while other muscles such as anterior deltoid, serratus anterior, trapezius and biceps brachii are also involved [3,7,8,9]. Traditionally, push-ups are performed on a flat stable surface at the width of the shoulders [10]; however, nowadays countless variations exist, aiming to isolate and produce different levels of impact on the muscles of interest [4,11]. Such variations include differences in hand position from standard to narrow or wide positions [12,13,14], suspension training systems [10,15], unstable surfaces [5,16] and different rotating handgrip devices [8,17]. Studies which have investigated and compared the electromyographic activation of the involved muscles found that their activation is much higher in more challenging conditions, such as on unstable surfaces and with the use of suspension devices compared to the traditional form of push-ups [5,10,15,16]. Even though the traditional form of push-ups is widely used, due to its simplicity [7], coaches, trainers and physical health practitioners are more interested in exercises that can induce higher muscle activation as this is associated with greater adaptation to strength, endurance and neuromuscular control over time [8,18].

An alternative and more practical variation of push-ups, challenging and capable of increasing muscle activation, is different hand positioning [9,13,14]. Different hand positioning on stable surfaces offers biomechanical advantages such as the ability to isolate specific muscles of interest and reduce variability from multidirectional instability from unstable surfaces and suspension systems, which often leads to misinterpretation of the results [19]. The most common variations in hand positions are the standard push-up with hands placed at shoulder width, the narrow base, also known as the diamond push-up, with hands placed under the chest forming a triangular shape on the floor with the fingertips and thumbs, and the wide push-up with hands placed wider than shoulder width, typically at 150% of the biacromial distance [7,14].

Several studies have investigated the influence of different hand position push-ups on the muscle activation of both the pectoralis major and triceps brachii; however, the findings are inconsistent. In the studies by Cogley et al. [14] and Kim et al. [13], both muscles had greater activation in the narrow compared to the wide hand placement push-up. Another study by Marcolin et al. [9] found that the narrow hand position provided the highest activation for both the pectoralis major and triceps brachii muscle, followed by the standard push-up, whereas the wide hand position provided the lowest activation. In contrast to the above studies, Allen et al. [17] found that during the wide push-up (in both conditions, with handgrip device and without), pectoralis major had the highest activity compared to triceps brachii, serratus anterior and posterior deltoid. Similarly, a study by Youdas et al. [8] found that during narrow hand placement the triceps brachii had the highest activation, while hand position (narrow, wide and standard) lacked a differentiating effect for pectoralis muscle. In the same study, the authors also found no significant differences between the traditional push-ups and push-ups performed with the use of handgrip devices, concluding that such devices should not be preferred when the goal is to strengthen the shoulder and arm muscles [8]. Furthermore, Nadzalan et al. [12] found that the pectoralis major had the highest activity during standard push-ups and the lowest during diamond push-ups, with the triceps muscle having the highest activity during the diamond push-ups and the lowest during the standard push-ups.

The controversy found in the literature on the electromyographic activation across push-ups with hand placement variations could be due to methodological aspects [11] which strongly influence the electromyographic (EMG) signal derived from the muscles. Such methodological aspects include self-selected [7] or controlled speed [8,9,14], the technique of performing the push-up [7,14], self-selected vs. standardized hand positioning [7,13,14], and normalization methods of the EMG signal [20] such as normalizing to maximal voluntary isometric contraction (MVIC) [8,9,14] versus using a task-specific EMG normalization technique [7,13]. In addition to the above, there is also limited information regarding the eccentric phase [9] of the push-up, as most of the studies are only focusing on the concentric phase (push-off) of the push-up.

Considering the controversy in the research and the inconsistency of the findings, the present study implemented a targeted methodological approach taking into account the possible anatomical variation between participants by allowing self-selected hand placement to minimize compensatory movement strategies and task-specific normalization techniques for the EMG signal to provide a more accurate representation of the muscle activation under the exact mechanical and neural demands of the task in both the concentric and eccentric phases. Thus, the aim of the present study was to investigate the electromyographic activation of the pectoralis major and triceps brachii muscles during the standard, diamond and wide hand position push-ups during both the eccentric and concentric phases of the movement. The hypotheses are that (a) triceps brachii will demonstrate the highest activation compared to pectoralis major, (b) push-ups from a narrow base will have higher muscle activation in both muscles and (c) the concentric phase will demonstrate the highest activation across push-up variations in both muscles.

## 2. Materials and Methods

### 2.1. Participants

Twenty healthy recreationally active males with 8 ± 2 h of training per week volunteered to participate in the present study (age: 22 ± 4 years, 176 ± 7 cm, 77 ± 12 kg). Participants were randomly recruited throughout the university via advertisements on social media and posters. Additionally, contact lists of participants from previous unrelated projects were utilized, along with snowball sampling, for further recruitment. The inclusion criteria for the participants were to be males, aged 18–35 years old, healthy based on their medical history, pain-free and able to perform push-up variants with proper technique. Participants who did not meet the inclusion criteria or had an injury and/or surgery on their shoulders, elbows, and wrists in the last five years were excluded from this study. Prior to the recruitment process, ethical approval was obtained from the Cyprus National Committee of Bioethics (CNBC, ΕΕΒΚ ΕΠ 2022.01.290). The study was conducted according to the international standards for the use of human subjects, as described within the Declaration of Helsinki [21]. All participants completed an informed consent form and the Physical Activity Readiness Questionnaire (PAR-Q) [22]. A post hoc analysis was performed in G*Power software (version 3.1.9.7) to compute the achieved power based on the given sample size of the group (N = 20), the number of measurements (3), and the calculated effect size (main effect of push-up variation) from partial *η^2^* of 0.399, based on the formula provided by Cohen [23], where *η^2^* is converted into Cohen’s *f* as $f=\sqrt{\frac{\eta 2}{1-\eta 2}}$ resulting in an effect size *f(U)* of 0.82 and an achieved power (1 − *β* error probability) of 1.00.

### 2.2. Study Design

A within-subject repeated-measures experimental design was used in the present study. Participants visited the laboratory once and performed the three push-up variations in a random order, while, simultaneously, their muscle electrical activity was recorded. The randomization order for the push-ups was generated for each participant using a free online tool (Randomizer.org; https://www.randomizer.org, accessed on 20 November 2024) prior to the measurements.

### 2.3. Instruments and Protocol

**Figure 1:**
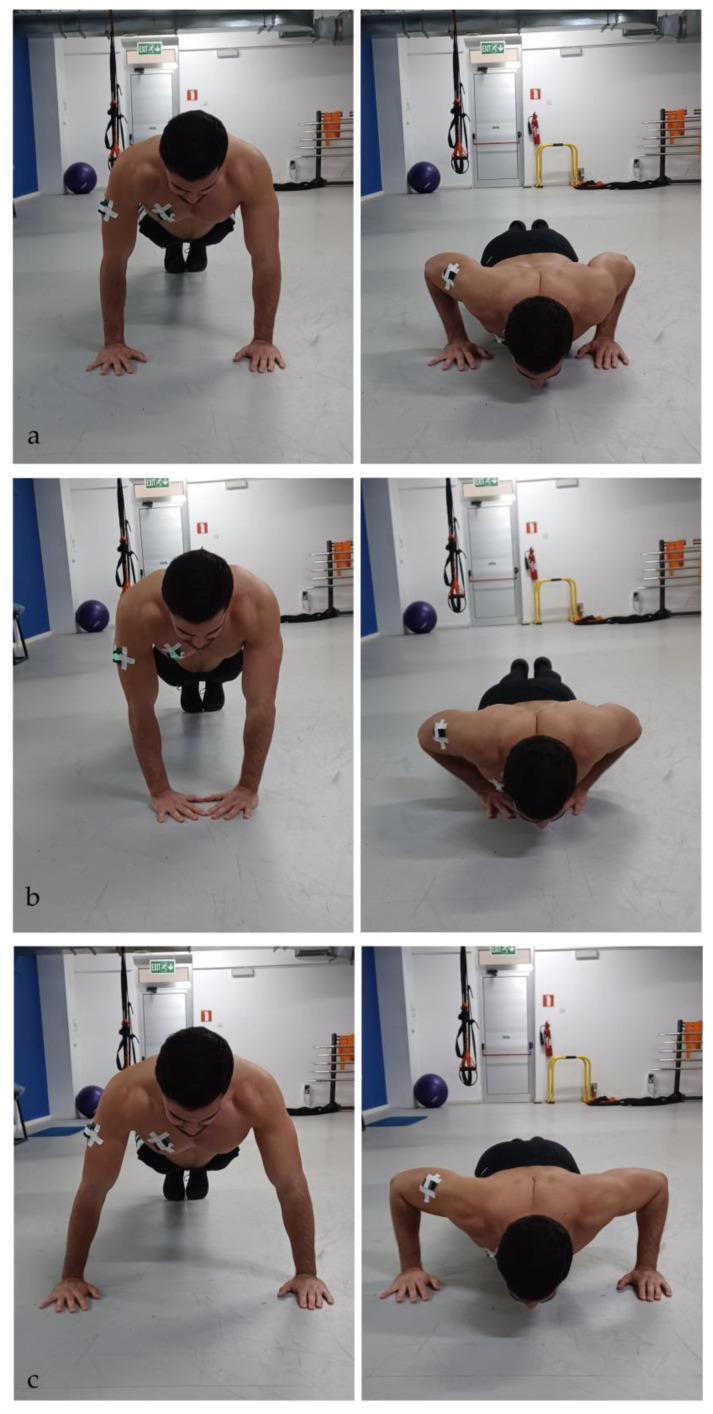
Standard push-up (**a**), diamond push-up (**b**), wide push-up (**c**); left figures: starting position, right pictures: end position. The EMG sensors were placed on the triceps brachii (lateral head) and on the pectoralis major (sternal head) and secured with additional tape to prevent them from falling off.

Participants performed a standardized warm-up using two multi-joint machine-based exercises (chest-press and pec-deck) of 12 repetitions each with light weights, with 2 min rest in between. After the warm-up, participants’ skin over the muscle belly of pectoralis major (sternal head) and triceps brachii (lateral head) on the dominant side was cleaned with alcohol and shaved. An electromyography sensor (Delsys Inc., Boston, MA, USA, Trigno Avanti Sensor) was placed parallel to the direction of the muscle fiber arrangement according to the SENIAM guidelines [24] and carefully secured to the skin with additional tape to prevent the sensors from falling off (Figure 1). To ensure consistency and accurate sensor placement, the operator was always the same. Participants were then instructed to perform the three push-ups (standard, diamond, wide) in a randomized order with 2 min of rest in between. The tempo was slow, resulting in 3 s of eccentric phase (elbow flexion) and 3 s of concentric phase (elbow extension) using verbal cues and controlled with a timer [14]. A test trial before each push-up variation was performed to evaluate the technique and correct it if necessary, and for the participants to get accustomed to the tempo of the movement. During the push-ups, the electrical activity of the muscles was recorded simultaneously. All push-ups started with the participants in a high plank position, forming a straight line from head to heel and with a verbal cue, the eccentric phase of the push-up (elbow flexion) was initiated. In all variations of push-ups, participants were instructed to maintain a straight body line by tightening their core with feet positioned together and lowering their body slightly above the floor and push-up to full extension with eyes focus ahead (Figure 1). For the standard push-up, participants were instructed to place their hands at shoulder width with their middle finger pointing forward and wrist aligned with their elbows at approximately 90°. For the diamond push-up, the specific instructions given were to place their hands under their chest in the midline of their sternum, with their thumbs and index finger forming a diamond shape on the floor and slowly lowering their chest by flexing their elbows while keeping them close to their torso avoiding also shrugging of the shoulders. For the wide push-up, participants were asked to place their hands in a self-selected, wider-than-shoulder-width position, that was comfortable for them and in which they were able to perform it in a correct form while avoiding compensatory movements.

### 2.4. Outcome Variables and Data Analysis

The EMG data were collected during the measurement using the Delsys EMG Trigno Wireless system (Delsys Inc., Boston, MA, USA), which was connected to a PC running Delsys EMGworks Acquisition software (version 4.8.0) and processed for further analysis using the software Delsys EMGworks Analysis (version 4.7.3.0) with an analog band-pass Butterworth fourth-order filter at 20–450 Hz and a sampling frequency of 1259.26 Hz [25]. The system also features a common mode rejection ratio > 80 dB and an input noise level < 0.75 µV RMS, providing a high signal-to-noise ratio and ensuring reliable EMG acquisition. The root mean square (RMS, mV) computations were performed for each repetition using a window length of 0.125 s with 0.0625 s of overlap. The specific window length, which corresponds to the default configuration of Delsys software, is further supported by the study of Burden et al. [26], where they found that window lengths between 0.1 and 0.25 s with 50% overlap produce stable and reliable mean EMG values. To assess the consistency of the root mean square (RMS, mV) across repetitions in the present study, additional reliability analyses were performed [27,28,29]. As the evaluation of consistency was not the primary focus of this study, the specific results are reported in the Methods section to support the methodological approach used.

In the present study, a task-specific EMG normalization technique was used [20,30,31,32]. For each push-up variation, the repetition that had the highest RMS value out of the six repetitions was used for normalization purposes and the other five repetitions were averaged into one value representing the RMS value of a push-up. To calculate the normalized to peak RMS (%), the average RMS value was normalized to the highest RMS value with the following equation: $\mathrm{Normalised}\mathrm{to}\mathrm{peak}\mathrm{RMS}\;\left(\%\right)=\left(\frac{{\mathrm{Mean}\mathrm{RMS}}_{\;\mathrm{Reps}\;1–5}}{{\mathrm{Max}\mathrm{RMS}}_{\;\mathrm{Reps}\;1–6}}\right)\times \;100$. The main outcome variables were the RMS (mV) and normalized to peak RMS (%) of each muscle during the concentric phase (elbow flexion) and eccentric phase (elbow extension) of the push-ups.

### 2.5. Consistency Between Repetitions

Participants performed six repetitions for each push-up variation, where the repetition exhibiting the highest RMS value was used as a reference for normalization, and the other five were averaged into one value representing the RMS of the specific push-up. For the evaluation of the consistency, only the five repetitions were used, excluding the repetition with the highest RMS value. The consistency of the repetitions between each variation in push-up, muscle and contraction phase was evaluated by means of intra-class coefficient analysis (ICC, 3,1) with a 95% confidence interval (CI: 95%). The specific ICC (3,1) model was selected because all measurements were obtained using the same fixed EMG setup within a single session, and consistency was evaluated only for individual repetitions and not for averaged values [33]. An ICC value of ≤0.50 was considered low, an ICC value of 0.50 to 0.75 was considered moderate, an ICC value of ≥0.75 was considered good, and an ICC value of ≥0.90 was considered excellent [28]. Coefficient of variation (CV, %) was calculated for each participant to quantify the variability relative to the mean with the equation $\mathrm{CV}=\left(\frac{\mathrm{SD}(5\mathrm{rep}.)}{\mathrm{Mean}(5\mathrm{rep}.)}\right)\times 100$, and was expressed as a percentage [27]. The individual values of CV (%) were then averaged and reported as mean and standard deviation (SD). Additionally, to provide an estimate of the precision of measurement, the standard error of measurement (SEM, mV) was calculated using the following equation: SEM = SD × √(1 − ICC) [27]. SEM was further used to calculate the minimal detectable change (MDC, mV), which is the minimal amount of change that a measurement must show to be greater than the within-subject variability and measurement error, calculated according to the following equation: MDC = 1.96 × SEM × √2 [29]. Both SEM and MDC are expressed in the units of measurements of the variable assessed. The outcome values are presented in Table 1, demonstrating excellent consistency based on the ICC values, for both the pectoralis major and triceps brachii muscles in both eccentric and concentric phases. However, during the concentric phase, the pectoralis major showed slightly higher variability compared to the triceps brachii muscle. The triceps brachii demonstrated higher consistency than the pectoralis major muscle, with lower CV (%), SEM (mV), and MDC (mV) values, reflecting a greater measurement precision. Among the three push-up variations, the diamond push-up yielded the lowest CV (%) values, especially for the triceps brachii muscle. Overall, the findings demonstrated a strong consistency of RMS (mV) measurements within individuals across repetitions, confirming consistent muscle activation patterns for each participant under each push-up variation. These reliability metrics were used solely to characterize within-condition measurement consistency and to support the methodological approach of the study.

### 2.6. Statistics

The data were initially tested for normal distribution with the use of the Shapiro–Wilk test. All variables met the assumption of normality (*p* > 0.05) and descriptive statistics were used to calculate mean and standard deviation for each dependent variable. To evaluate the difference in RMS (mV) and normalized to peak RMS (%) between the pectoralis major and triceps brachii muscles, and the two contraction phases (eccentric: elbow flexion, concentric: elbow extension) during the three variations of push-ups (diamond, standard, wide), two separate three-way ANOVA tests (for each dependent variable) were used with a Bonferroni post hoc correction. Mauchly’s test was used to evaluate the assumption of sphericity; when this assumption was not met, the Greenhouse–Geisser correction was applied. The main effects of muscle, contraction phase, push-up variation, and their interaction from the three-way ANOVAs were interpreted using an alpha level of 0.05. In addition, effect sizes for post hoc pairwise comparisons were calculated using Cohen’s *d* [23]. Cohen’s *d* was calculated from the *t*-value, which was computed as the mean difference divided by the standard error of each comparison (*t* = mean diff/SE) and adjusted by dividing it by the square root of the sample size (n = 20) [34]. According to Cohen, a size of 0.2 to 0.49 indicates a small effect, 0.5 to 0.79 indicates a moderate effect, and above 0.80 a large effect [23]. All statistical analyses were performed using SPSS (SPSS Statistics 26, IBM, Armonk, NY, USA) and Microsoft Excel 365 (version 2506, Microsoft Corporation, Redmond, WA, USA).

## 3. Results

**Figure 2:**
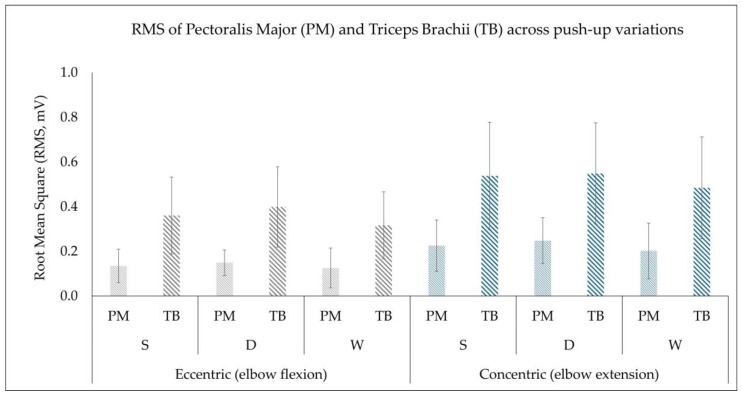
Root mean square (RMS, mV) of the pectoralis major (PM) and triceps brachii (TB) muscles presented as mean ± standard deviation (error bars) during the eccentric phase (elbow flexion) and concentric phase (elbow extension) across the three push-up variations (standard (S), diamond (D), and wide (W)).

**Figure 3:**
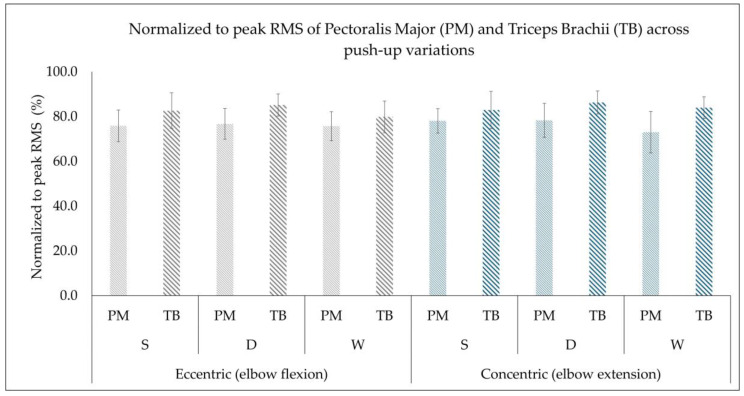
Normalized to peak RMS (%) of the pectoralis major (PM) and triceps brachii (TB) muscles presented as mean ± standard deviation (error bars) during the eccentric phase (elbow flexion) and concentric phase (elbow extension) across the three push-up variations (standard (S), diamond (D), and wide (W)).

The descriptive data are presented in Figure 2 and Figure 3 as mean ± standard deviation for both variables, RMS (mV) and normalized to peak RMS (%) for pectoralis major and triceps brachii muscle, across the three variations in push-ups during both the eccentric (elbow flexion) and concentric phases (elbow extension).

### 3.1. Root Mean Square (RMS, mV)

There was a violation of sphericity for the main effect of push-up variation (*p* = 0.023) as indicated by Mauchly’s test and, thus, the Greenhouse–Geisser corrections (*ε* = 0.745) were applied. No other violation of sphericity was observed for muscles, contraction phase, and their interaction (*p* > 0.05).

A statistically significant main effect of muscle was observed, *F*_(1,19)_ = 86.015, *p* < 0.001, *η^2^* = 0.819, with the triceps brachii muscle demonstrating a higher RMS than the pectoralis major across all three push-up variations (*p* < 0.001, Cohen’s *d* = 2.08; very large effect). The contraction phase was also found to have a statistically significant effect, *F*_(1,19)_ = 96.308, *p* < 0.001, *η^2^* = 0.835, with the concentric phase producing a greater RMS than the eccentric phase (*p* < 0.001, Cohen’s *d* = 2.18; very large effect). A statistically significant main effect of push-up variation was also observed (Greenhouse–Geisser corrected), *F*_(1.489,28.297)_ = 12.599, *p* < 0.001, *η^2^* = 0.399. Pairwise comparisons revealed that both the diamond and standard push-up had higher RMS values than the wide push-up (*p* = 0.002 and *p* = 0.002, Cohen’s *d* = 0.86 and *d* = 0.92, respectively; large effect). However, no statistically significant difference was found between the diamond and standard push-ups (*p* = 0.146, Cohen’s *d* = 0.34; small effect).

A statistically significant interaction between muscle and contraction phase was observed, *F*_(1,19)_ = 29.224, *p* < 0.001, *η^2^* = 0.606. Post hoc analysis indicated that both muscles showed increased RMS during the concentric phase, with a greater relative increase in the triceps brachii compared to the pectoralis major muscle, highlighting the triceps brachii’s primary contribution to force generation during the concentric phase. Furthermore, no statistically significant interactions were found for muscle × push-up (*p* = 0.211) or contraction phase × push-up (*p* = 0.407), suggesting that the effects of push-up variation were consistent across muscles and contraction phases. The three-way interaction of muscle × contraction phase × push-up was not statistically significant (Greenhouse–Geisser corrected), *F*_(1.804,34.276)_ = 2.930, *p* = 0.072, *η^2^* = 0.134, indicating that contraction activation patterns (concentric/eccentric) were consistent across all push-up variations.

### 3.2. Normalized to Peak RMS (%)

No significant violation of sphericity according to Mauchly’s test was found for muscles, contraction phase, push-up variations and their interactions (*p* > 0.05), and thus no further corrections were applied for the normalized to peak RMS (%).

A statistically significant main effect was found for muscle (*F*_(1,19)_ = 56.26, *p* < 0.001, *η*^2^ = 0.748) with the pairwise comparison indicating a statistically significantly higher normalized to peak RMS (%) in triceps brachii compared to the pectoralis major muscle (*p* < 0.001, Cohen’s *d* = 1.68; very large effect size). The main effect of contraction phase (eccentric, concentric) was found to be not statistically significant (*F*_(1,19)_ = 1.729, *p* = 0.204, *η^2^* = 0.083, Cohen’s *d* = 0.28; small effect size), indicating that in both concentric and eccentric phases a similar proportion of maximal activation is required within each muscle. A statistically significant main effect of push-up variations was observed (*F*_(2,38)_ = 5.23, *p* = 0.010, *η^2^* = 0.216). Pairwise comparisons with Bonferroni adjustment showed statistically significantly higher normalized to peak RMS (%) in diamond push-ups compared to wide push-ups (*p* = 0.015, Cohen’s *d* = 0.71; moderate effect size). Standard push-ups also had statistically significantly higher normalized to peak RMS (%) compared to wide push-ups (*p* = 0.015, Cohen’s *d* = 0.44; small-to-moderate effect size). However, no statistically significant difference was found in normalized to peak RMS (%) when comparing diamond to standard push-up (*p* = 0.513, Cohen’s *d* = 0.32; small effect size). Thus, the above findings indicate that the narrower the hand position, the higher the muscle activation, especially for the triceps brachii muscle.

No statistically significant interactions (*p* > 0.05) were observed for muscle × contraction phase (*F*_(1,19)_ = 0.935, *p* = 0.346, *η^2^* = 0.047), muscle × push-up (*F*_(2,38)_ = 0.609, *p* = 0.549, *η^2^* = 0.031), contraction phase × push-up (*F*_(2,38)_ = 0.050, *p* = 0.951, *η^2^* = 0.003), and muscle × contraction phase × push-up (*F*_(2,38)_ = 2.638, *p* = 0.085, *η^2^* = 0.122). These findings suggest that while push-up variations can influence the level of normalized muscle activation, the pattern of relative muscle recruitment remained consistent across contraction phases as both muscles maintained a similar proportion of activation during concentric and eccentric phases.

## 4. Discussion

The aim of the present study was to investigate and compare the electromyographic activation of the pectoralis major and triceps brachii muscles during the three hand position variations in push-ups: the standard, the diamond, and the wide. The main outcome variables were the RMS (mV) and the normalized to peak RMS (%) of pectoralis major and triceps brachii muscles calculated in both contraction phases (eccentric and concentric) of each push-up. The main findings revealed that the triceps brachii indeed had the highest activation compared to the pectoralis major especially during the diamond push-up, followed by the standard and then the wide with the least of activation, thus confirming the initial hypothesis. In addition, the concentric phase demonstrated the highest RMS (mV) activation in both muscles in all three variations, confirming the hypothesis. However, when normalizing to peak RMS (%), no differences were observed between eccentric and concentric phases, indicating a consistency of muscle effort, reflecting a similar ability to sustain near-maximal activation regardless of hand position push-up variation.

In the present study, the two muscles were chosen specifically as the pectoralis major is responsible for horizontal and diagonal adduction, along with internal rotation of the humerus, and the triceps brachii is responsible for elbow extension. Both are considered as primary acting muscles during the push-up exercise [7,8]. However, due to the anatomical function of the pectoralis muscle, one would expect higher activation in the wide and less in diamond [14]. Some authors have indeed made such a hypothesis before [14], with their findings, however, being aligned with the findings of the present study and that of others, with the pectoralis muscle being more activated in the diamond push-up and the least activated during the wide push-up [9,13,14]. A possible explanation is the influence of elbow positioning during the specific push-ups [35,36]. In the diamond push-up, the elbow remains tucked close to the torso, increasing shoulder horizontal adduction while keeping the humerus close to midline. This position not only activates the triceps brachii more; it also causes the pectoralis, especially its sternal fibers, to work even harder, assisting in moving the arms towards the midline under the load. Meanwhile, during the wide push-ups, the elbow flares outwards, placing the humerus in greater abduction and reducing the range and torque demands of horizontal adduction, and thus the pectoralis works in a more shortened position, reducing its mechanical efficiency and shifting the activation toward anterior deltoid and shoulder stabilizer muscles [35,36].

Conversely, there are studies in which their findings are not in agreement with the above, leading to a controversy in the literature [8,12,17]. Allen et al. [17] found that during the wide push-ups the pectoralis exhibited a significantly higher activation compared to the triceps, whereas, in our findings, even in the wide push-up, the triceps brachii had statistically significantly higher activation compared to the pectoralis muscle. Youdas et al. [8] partially agree with the findings of the present study, as they have found higher activation of the triceps brachii in the narrow hand position; however, they lacked a differentiating effect for hand position (narrow, wide and standard) for pectoralis major muscle, suggesting that its activation remained constant through the three push-up variations. Another study which adds to the controversy in the literature and disagrees to a great extent with the present study is the study by Nadzalan et al. [12]. In that specific study, they found pectoralis major to have the highest activity during standard push-ups and the lowest during diamond push-ups, whereas the triceps brachii muscle was found to have the highest activation in diamond and the lowest in standard push-ups [12].

Previous studies have standardized the hand position during wide push-ups based on the biacromial distance to set a defined width position at 150% of the shoulder width [7,13]. In this study, the width of the hands, for the wide push-ups, was not standardized as fixed positions may place participants with different anatomical structures into mechanically disadvantageous joint angles leading to compensatory movements, altering the electrical activation of the muscles [37]. Thus, by allowing self-selected hand width [38], variability associated with anatomical differences can be reduced and movement execution can reflect more closely real training and rehabilitation settings. While this approach may limit direct comparison between studies and might be perceived as a possible study limitation, it enhances the relevance and applicability of the findings, as coaches and health professionals commonly allow individuals to adopt a comfortable, self-selected hand position that aligns with functional training and rehabilitation practices [39,40].

In the present study, both contraction phases of the push-ups, eccentric (elbow flexion) and concentric (elbow extension), were investigated. When comparing the RMS (mV), the concentric phase produced the highest activation in both muscles in all three push-up variations compared to the eccentric phase. This finding agrees with the study by Marcolin et al. [9] where they found that during the ascendant phase (concentric, elbow extension), the electrical activity of the muscles, including also the pectoralis major and triceps brachii, was statistically significantly higher compared to the descendant phase (eccentric, elbow flexion). However, when the RMS signal was normalized to the peak activation within each condition (%), no significant differences were found, indicating that the effort produced by the muscles in both contraction phases was consistent and stable, reflecting a similar ability to sustain near-maximal activation throughout the set. This finding might be of great interest to trainers and coaches, as both concentric as well as eccentric training produce specific neuromuscular adaptations that enhance shoulder performance, joint stability, and injury prevention with important implications in rehabilitation as well [4,41,42]. Eccentric exercises are characterized by greater mechanical tension, inducing specific neuromuscular adaptations, promoting muscle hypertrophy [43] and enhancing tendon adaptation [44,45], while concentric exercises contribute to force production and functional movement capacity [46]. Comparable muscle activation across both phases of push-ups may enhance joint stability, aiding in the risk reduction for overuse injuries, which is a possible implication for rehabilitation and exercise [1,4].

Normalization of the EMG involves rescaling the data from microvolts (mV) to a percentage of a reference value obtained [31]. The gold-standard technique is using the RMS (mV) derived from MVIC as a reference value [20]. However, when it comes to dynamic movements, aspects such as differences in muscle action, load, and velocity of the dynamic activity versus MVIC raise concerns about its appropriateness [31]. Thus, task-specific normalization methods for dynamic activities using the maximal value recorded during the actual task under the same biomechanical and neural conditions have been proposed but are rarely used [20,30,31,32]. Most previous relevant studies [8,10,12,14,17] have used MVIC for normalization purposes, and only a couple of studies have used task-specific normalization techniques, considering as a reference for normalization the RMS from the standard push-up [7,13]. In this study, the normalization technique used was also task-specific, with the exception that the repetition with the highest RMS value from each push-up variation, in each contraction phase and in each muscle was normalized to its average value. This approach also allowed the investigation of the within-phase consistency by expressing each repetition RMS relative to its peak, providing deeper insights, especially into the contraction phases, eccentric and concentric, of the push-up. In addition, the influence of muscle velocity, changes in length and contraction regimen are inherently accounted for by using this specific approach, whereas with the MVIC normalization approach, this is not possible [31].

A possible limitation of the present study is the fact that the range of motion, an aspect that influences muscle activation, was not recorded. Even though participants were instructed on how to perform each push-up in terms of technique and speed, small variations probably existed between participants, which the authors were unable to evaluate. In addition, only male recreationally active participants were included, and thereby generalizing the findings in untrained or especially in highly trained male individuals [47] might not be applicable as elite athletes exhibit greater neuromuscular efficiency and different muscle activation strategies. Furthermore, the inclusion of only males restricts also its applicability to females, as sex-specific neuromuscular differences such as muscle fiber composition, hormonal influences and fatigue resistance may have a different impact on muscle activation patterns [48,49]. Another possible limitation is the number of muscles assessed. In the present study, the authors had the capacity to include only two muscles and based on the literature the pectoralis major and triceps brachii are the prime muscles during push-ups [7,8]. However, for a more comprehensive investigation, adding also the synergist and stabilizer muscles such as the anterior deltoid, trapezius and serratus anterior would have provided a more complete understanding of the electromyographic activation patterns and muscular demands during push-ups with varied hand positioning.

## 5. Conclusions

Push-ups of varied hand positions can strategically target specific muscles, thus enhancing strength and functional performance. The diamond push-up elicited the highest activation for both the triceps brachii and pectoralis major muscles followed by the standard, while the wide push-up produced comparatively the lowest activation. The triceps brachii muscle was activated the most in all three push-up variations. The concentric phase produced the highest RMS activation in both muscles across the three push-up variations. However, when RMS values were normalized to their task-specific peak RMS, a consistent ability to sustain a near-maximal activation was observed in both muscles, contraction phases and push-up variations. These findings suggest that although a specific hand position can influence the amount of muscle activation, all three hand position variations of push-ups are equally effective at sustaining a high muscle activation across repetitions. This has important applications in strength training and rehabilitation contexts as it provides trainers and healthcare practitioners with flexibility when selecting push-up variations while maintaining high levels of muscle activation.

## Figures and Tables

**Table 1:** Consistency of root mean square (RMS, mV) between repetitions for each contraction phase, muscle and push-up.

Contraction Phase	Muscles	Push-Up	ICC (3,1)	CV (%)	SEM (mV)	MDC (mV)
Eccentric (elbow flexion)	Pectoralis	Standard	0.976	16 ± 8	0.003	0.009
		Diamond	0.965	13 ± 4	0.004	0.011
		Wide	0.979	15 ± 8	0.003	0.008
	Triceps Brachii	Standard	0.988	11 ± 5	0.004	0.011
		Diamond	0.991	9 ± 3	0.003	0.009
		Wide	0.986	11 ± 5	0.004	0.011
Concentric (elbow extension)	Pectoralis	Standard	0.978	15 ± 6	0.005	0.014
		Diamond	0.964	16 ± 6	0.007	0.020
		Wide	0.976	17 ± 8	0.005	0.015
	Triceps Brachii	Standard	0.988	11 ± 5	0.006	0.016
		Diamond	0.988	9 ± 4	0.005	0.014
		Wide	0.990	10 ± 4	0.004	0.012

Measures of consistency: ICC = intra-class correlation coefficient, CV = coefficient of variation (mean ± SD); SEM = standard error of measurement; MDC = minimal detectable change.

## Data Availability

The data are available from the corresponding author upon reasonable request.
